# A Rare Combination of von Willebrand Disease Type 2A and 2M: Diagnostic and Therapeutic Challenges – A case report

**DOI:** 10.1016/j.rpth.2026.106867

**Published:** 2026-07-16

**Authors:** Floor Derikx, Inge Merry, Dennis Willemsen, Erik Beckers, Yvonne Henskens, Floor Heubel-Moenen

**Affiliations:** 1 Department of Internal Medicine-Hematology, Maastricht University Medical Center+, Maastricht, The Netherlands; 2 Department of Internal Medicine, Anna Ziekenhuis, Geldrop, The Netherlands; 3 Central Diagnostic Laboratory, Maastricht University Medical Centre+, Maastricht, The Netherlands; 4 Cardiovascular Research Institute Maastricht (CARIM), Maastricht University, The Netherlands

**Keywords:** bleeding disorders, genetic testing, hemostasis, multidisciplinary approach, type 2A/2M, von Willebrand disease

## Abstract

**Background:**

Combined von Willebrand disease (VWD) type 2A/2M is a rare phenotype characterized by overlapping qualitative defects affecting both multimer structure and von Willebrand factor (VWF) function.

**Key Clinical Question:**

How can congenital VWD be distinguished from acquired von Willebrand syndrome (AVWS) when both conditions contribute to an abnormal laboratory phenotype?

**Clinical Approach:**

A 73-year-old man with a previous diagnosis of VWD type 2M presented with severe gastrointestinal bleeding and severe aortic stenosis. Repeat VWF analysis showed loss of high-molecular-weight multimers, consistent with AVWS secondary to shear stress. Treatment with recombinant VWF (Veyvondi) restored high-molecular-weight multimers and achieved hemostatic control. Following transcatheter aortic valve implantation, the abnormal multimer pattern persisted. Subsequent genetic testing identified a pathogenic *VWF* variant, p.Leu1446Pro, confirming congenital VWD type 2A/2M.

**Conclusion:**

This case demonstrates how AVWS may obscure an underlying congenital VWD phenotype. Combined phenotypic and molecular diagnostics are essential for accurate classification, therapeutic decision making, and interpretation of complex VWD phenotypes.

## Essentials

• A rare von Willebrand disease subtype can be difficult to diagnose accurately.
• We describe a 73-year-old man with bleeding, aortic valve stenosis, and von Willebrand disease.
• Acquired von Willebrand syndrome masked an underlying inherited bleeding disorder.
• Genetic testing enabled the correct diagnosis and guided treatment decisions.

## 1 Introduction

von Willebrand disease (VWD) is the most common inherited bleeding disorder, caused by quantitative or qualitative defects in the von Willebrand factor (VWF) protein [1]. Type 2 variants comprise 4 qualitative types: 2A, 2B, 2M, and 2N, each reflecting distinct structural and functional abnormalities. The coexistence of defects affecting both type 2A and type 2M mechanisms can result in a complex clinical presentation, characterized by overlapping features of both defects. We report a patient with combined congenital VWD type 2A/2M, illustrating the diagnostic and therapeutic challenges of overlapping mechanisms.

## 2 Case Description

A 73-year-old man was transferred from another hospital because of profound hypovolemic shock due to rectal bleeding and marked peripheral edema of the lower limbs. His medical history included hypertension, type 2 diabetes mellitus, and VWD type 2M, previously diagnosed based on a reduced VWF activity (Act)-to-antigen (Ag) ratio (VWF:Act 24 IU/dL; VWF:Ag 58 IU/dL; VWF:Act/Ag ratio 0.4; factor [F]VIII 92 IU/dL) in combination with a normal VWF multimer pattern. Family history was notable for a sister with a diagnosis of VWD, although no phenotypic or genetic characterization was available. The patient’s father and paternal aunt were also reported to have an increased bleeding tendency, but no formal diagnosis of VWD had been established. The patient was treated with VWF concentrate and received multiple blood transfusions, after which the bleeding ceased clinically.

Endoscopic evaluation revealed no clear bleeding focus. Upon physical examination, however, a systolic murmur was detected, consistent with aortic valve stenosis. Transthoracic echocardiography confirmed severe aortic stenosis. The reanalysis of VWF again showed a decreased VWF activity-to-antigen ratio (VWF:Act 34 IU/dL; VWF:Ag 74 IU/dL; ratio 0.5). However, VWF multimer analysis now demonstrated a loss of high-molecular-weight multimers (HMWMs; Figure A) [2], suggesting an acquired type 2A von Willebrand syndrome (VWS) caused by the aortic stenosis. This led to the differential diagnosis of gastrointestinal bleeding secondary to angiodysplasia related to aortic stenosis, known as Heyde’s syndrome.

**Figure:**
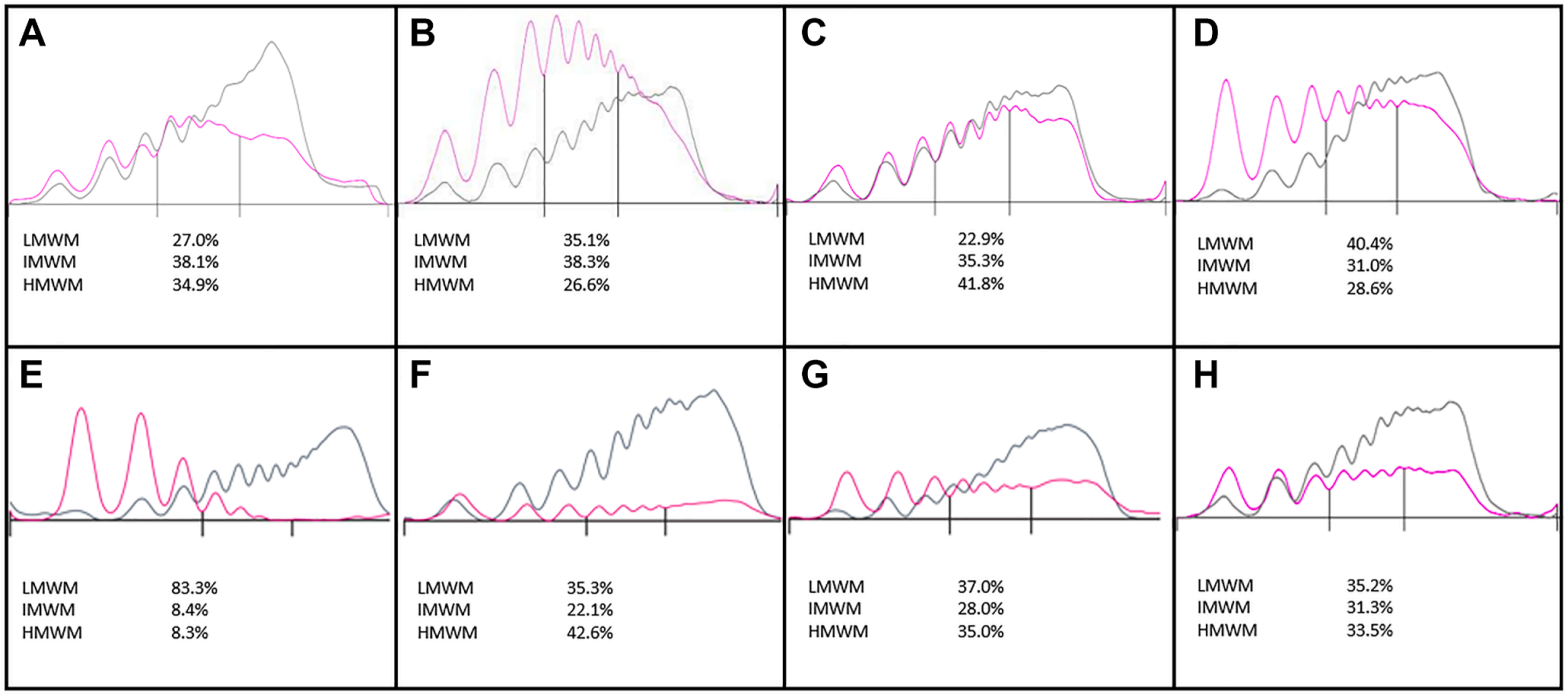
(A–D) von Willebrand factor multimer patterns of the case at different time points and (E–H) comparison of multimer patterns of different von Willebrand disease (VWD) types with those of the case. (A) Multimer pattern of the case upon hospital admission. (B) Multimer pattern of the case after administration of Wilfactin. (C) Multimer pattern of the case after administration of Veyvondi. (D) Multimer analysis 1 month following transcatheter aortic valve implantation procedure. (E) Classic VWD type 2A with loss of high-molecular-weight multimers (HMWMs) [2]. (F) Type 2M with normal multimeric pattern [2]. (G) Overlap between type 2A and 2M with mild reduction of HMWMs [2]. (H) Multimeric pattern of the case 6 months after transcatheter aortic valve implantation procedure. Each panel illustrates the fractions (percentage) of von Willebrand factor multimers by molecular weight (low-molecular-weight multimers [LMWMs], intermediate-molecular-weight multimers [IMWMs], and HMWMs) based on their electrophoretic mobility. Black lines represent control values, and pink lines represent patient multimers. *Source:* Panels E–G are reproduced from Seidizadeh et al. [3] with permission.

### 2.1 Heyde’s syndrome

Heyde’s syndrome describes the association between aortic stenosis and gastrointestinal bleeding, most commonly caused by angiodysplasia [4,5]. In 67% to 92% of patients with aortic stenosis, acquired type 2A VWS is present, with the severity of the VWS strongly correlating with the degree of valvular stenosis [6]. VWF is a multimeric glycoprotein (GP) essential for platelet adhesion and aggregation. Its functional activity largely depends on the presence of HMWMs, which contain most of the binding sites for platelets and collagen. Loss of these multimers may therefore lead to an increased bleeding tendency. The underlying mechanism of acquired type 2A VWS is closely linked to the hemodynamic changes occurring in a narrowed aortic valve. Under physiological conditions, circulating VWF multimers adopt a globular, folded conformation that conceals the proteolytic cleavage site [7]. In severe aortic stenosis, blood must flow through the narrowed valve at increased velocity, resulting in turbulent flow and high shear stress. When these multimers pass through the stenotic valve, extreme shear forces mechanically unfold and elongate them, exposing the Tyr1605–Met1606 bond within the A2 domain, the cleavage site recognized by ADAMTS-13 [8]. This increased proteolysis leads to the selective loss of the largest VWF multimers from the circulation [9]. As a result, an acquired type 2A VWS develops, predisposing to bleeding, particularly from the gastrointestinal mucosa [10]. Chronic mucosal hypoperfusion and altered hemodynamics contribute to the development of angiodysplasia. Furthermore, the absence of HMWMs may promote angiogenesis through multiple VWF-dependent pathways. In addition to dysregulation of VEGFR-2 signaling, VWF deficiency has been associated with altered angiopoietin-2/Tie-2 signaling, impaired endothelial homeostasis, and abnormal vascular remodeling. These mechanisms may contribute to the development of fragile vascular malformations and angiodysplasia, thereby increasing the risk of gastrointestinal bleeding [[11], [12], [13]]. In most patients, aortic valve replacement leads to rapid normalization of the VWF multimer distribution and correction of the acquired hemostatic defect. Persistent abnormalities after valve replacement should prompt consideration of an underlying congenital VWF defect [5]. Based on the multidisciplinary diagnostic findings, transcatheter aortic valve implantation (TAVI) was considered indicated in this patient.

### 2.2 Clinical course

Notably, despite a lifelong bleeding tendency and a previous diagnosis of VWD, the patient had never experienced severe gastrointestinal bleeding before the development of severe aortic stenosis. His bleeding phenotype predominantly consisted of epistaxis, easy bruising, and bleeding after dental procedures and surgery. The onset of severe gastrointestinal bleeding coincided with the development of aortic stenosis and the acquired loss of high-molecular-weight VWF multimers. During hospitalization, the patient was treated for gastrointestinal bleeding with a VWF concentrate containing a VWF:FVIII ratio >10 given his already elevated FVIII level. In The Netherlands, 2 VWF products with such a ratio are currently available: Wilfactin (LFB Biomédicaments) (plasma-derived) and Veyvondi (Takeda Nederland BV) (recombinant vonicog alfa). Initial treatment with Wilfactin was clinically effective but did not result in restoration of HMWMs on VWF multimer analysis (Figure B). After the diagnosis of aortic stenosis and the clinical suspicion of Heyde’s syndrome, therapy was switched to Veyvondi. Unlike Wilfactin, Veyvondi contains a higher proportion of HMWMs and ultralarge multimers, which are crucial for primary hemostasis under high shear stress, such as in the gastrointestinal vascular bed [2]. A subanalysis by Leebeek et al. [14] of a multicenter, open-label phase III trial investigated on-demand bleeding treatment in patients with severe VWD using Veyvondi. Fifteen gastrointestinal bleeds in 11 patients were analyzed, with a 93% success rate. Treatment was well tolerated and resulted in rapid and sustained hemostasis, even in previously refractory cases. In our patient, administration of Veyvondi led to normalization of the multimeric pattern (Figure C), consistent with improved correction of the underlying defect. Prior to the TAVI procedure, Veyvondi was administered again according to a predefined protocol, and the intervention proceeded uneventfully, without bleeding complications. After successful TAVI, the VWF multimer pattern was re-evaluated. The intervention was expected to normalize the multimer distribution through reduction of shear stress; however, this was not observed. The postoperative analysis still showed a reduction in HMWMs (Figure D). To clarify this discrepancy, genetic testing was performed. Genetic analysis revealed that the patient carried not only VWD type 2M, as initially suspected, but also a rare combined VWD type 2A/2M, caused by a heterozygous pathogenic variant in the *VWF* gene: p.Leu1446Pro.

### 2.3 VWD type 2A/2M

In classical VWD type 2A, HMWMs are lost due to increased proteolysis by ADAMTS-13, whereas in type 2M, HMWMs are preserved, but VWF exhibits defective binding to GPIb on platelets or to collagen. Previous studies have described VWD variants with overlapping features of types 2A and 2M, particularly the p.Arg1374Cys variant, which has been considered a challenge to the traditional classification because it combines impaired platelet-dependent function with subtle multimeric abnormalities [15]. The present case extends these observations by demonstrating how superimposed acquired VWS due to severe aortic stenosis can further obscure the underlying phenotype and complicate diagnostic interpretation. VWD type 2A/2M is characterized by dual structural abnormalities affecting both multimer stability and platelet binding. The pathogenic variant in our patient impairs VWF function and stability, leading to defective GPIb interaction and loss of HMWMs, features typical of both types. A recent study by Seidizadeh et al. [3] demonstrated that patients carrying the *VWF* variants p.Arg1315Leu, p.Arg1374Cys, and p.Arg1374His exhibit this dual phenotype. These variants are located at the interface between the A1 and A2 domains of VWF, regions critical for platelet GPIbα binding (A1 domain) and proteolytic cleavage by ADAMTS-13 (A2 domain), and are generally inherited in an autosomal dominant manner. Structural modeling revealed that p.Arg1315Leu induces a compact, less flexible conformation, whereas p.Arg1374His and p.Arg1374Cys result in elongated structures. These conformational alterations disrupt both platelet binding and multimer stability in plasma. Clinically, these mutations are associated with markedly decreased VWF:Act/VWF:Ag ratios, reflecting impaired platelet binding (type 2M), and mildly reduced VWF:collagen binding/VWF:Ag ratios, indicating partial loss of collagen binding and HMWMs (type 2A). The multimeric profiles of affected patients showed a decrease in HMWMs and an increase in intermediate- and low-molecular-weight multimers, a dual phenotype not captured by existing subtype definitions (Figure E, type 2A; Figure F, type 2M). Our patient’s multimeric pattern closely resembled that reported by Seidizadeh et al. [3] (Figure G, H), supporting the diagnosis of VWD type 2A/2M. The mutation found in our patient, p.Leu1446Pro, has previously been described only once by Casonato et al. [16]. This mutation, located in the A3 domain of VWF, primarily responsible for collagen binding, also affects multimer assembly and protein stability. In the original study, this variant produced a complex phenotype with reduced VWF synthesis, abnormal multimer distribution with loss of HMWMs, and markedly decreased VWF activity despite normal VWF:Ag and FVIII:concentration responses to desmopressin. The multimer pattern improved only partially, with persistent overrepresentation of smaller multimers. Expression of the mutant protein in baby hamster kidney cells confirmed reduced synthesis and secretion and replicated the multimer defect, demonstrating that the mutation alone was sufficient to cause the combined phenotype. Interestingly, the multimer pattern observed after TAVI showed only a mild persistent reduction in HMWMs, closely resembling the recently described type 2M/2A phenotype [3]. In contrast, the multimer pattern at hospital presentation demonstrated a more pronounced loss of HMWMs, likely reflecting the superimposed acquired VWS caused by severe aortic stenosis. Because historical multimer analyses from the time of the original diagnosis were not available for re-evaluation, it cannot be excluded that a subtle congenital type 2M/2A phenotype was initially interpreted as type 2M according to the classification criteria available at that time. Although speculative, age-related changes in posttranslational modifications of VWF may also influence multimer stability and susceptibility to proteolysis.

## 3 Conclusion

This case highlights the critical role of genetic testing in atypical or overlapping VWD variants. Molecular confirmation complements phenotypic analysis, refines classification, and guides individualized therapy. Importantly, this case demonstrates how acquired VWS secondary to aortic stenosis may obscure or mimic inherited VWD phenotypes and complicate diagnostic interpretation. Integrating clinical, laboratory, and molecular findings is therefore essential when phenotypic results appear discordant. In combined forms such as VWD type 2A/2M, this comprehensive approach enables accurate classification and personalized treatment strategies [17,18].
